# Case of Rupture of the Tendon Achilles by Muscular Effort

**Published:** 1852-06

**Authors:** S. Barrett

**Affiliations:** Le Roy, Genesee co., N. Y.


### ART. II. — *Case of Rupture of the Tendon Achilles by Muscular Effort.*

By S. Barrett, M. D., Le Roy, Genesee co., N. Y.

I send you the following, to me, unique case of Rupture of the Tendon Achilles, by muscular effort:

J. H., laborer, aged 48, of intemperate habits, while walking, in Nov. last, across a bridge, stepped upon the edge of a hole with his right foot, letting the heel drop into it; and making a sudden effort to recover himself, he felt a sharp pain extending from the heel up the leg, accompanied by an audible snap. In attempting to go a few steps farther he felt the same again, which rendered him unable to walk. He was brought home, and on examination the Tendon Achilles was found entirely separated about two inches above its insertion. The divided extremities were nearly an inch apart, as plainly to be felt as after a sub-cutaneous section for club-foot. He has been unable to walk without a crutch or cane until within the last month, but at the date of this the union seems to be pretty firm, and he walks pretty well by favoring it a little.

Le Roy, Genesee co., N. Y., April 24, 1852.

